# Polymerization Kinetics of Cyanate Ester Confined to Hydrophilic Nanopores of Silica Colloidal Crystals with Different Surface-Grafted Groups

**DOI:** 10.3390/polym12102329

**Published:** 2020-10-12

**Authors:** Andrey Galukhin, Guzel Taimova, Roman Nosov, Tatsiana Liavitskaya, Sergey Vyazovkin

**Affiliations:** 1 Alexander Butlerov Institute of Chemistry, Kazan Federal University, Kremlevskaya Str. 18, 420008 Kazan, Russia; taimowa17@mail.ru (G.T.); romanosow@mail.ru (R.N.); 2 Department of Chemistry, University of Alabama at Birmingham, 901 S. 14th Street, Birmingham, AL 35294, USA; tliavi@uab.edu

**Keywords:** cyanate esters, polymerization kinetics, confinement, colloidal crystals, thermal analysis, isoconversional kinetic analysis

## Abstract

This study investigates the kinetics of confined polymerization of bisphenol E cyanate ester in the nanopores of the three types of silica colloidal crystals that differ in the concentration and acidity of the surface-grafted proton-donor groups. In all three types of pores, the polymerization has released less heat and demonstrated a very similar significant acceleration as compared to the bulk process. Isoconversional kinetic analysis of the differential scanning calorimetry measurements has revealed that the confinement causes not only a dramatic change in the Arrhenius parameters, but also in the reaction model of the polymerization process. The obtained results have been explained by the active role of the silica surface that can adsorb the residual phenols and immobilize intermediate iminocarbonate products by reaction of the monomer molecules with the surface silanols. The observed acceleration has been quantified by introducing a new isoconversional-isothermal acceleration factor *Z_α_*_,*T*_ that affords comparing the process rates at respectively identical conversions and temperatures. In accord with this factor, the confined polymerization is 15–30 times faster than that in bulk.

## 1. Introduction

Exploring chemical reactions under confinement, e.g., inside nanopores [1,2,3], nanobrushes [4], or self-assembled monolayers [5,6,7,8], has been of major research interest in the areas of catalysis, polymer, supramolecular, and biochemistry. Studying the effects of confinement on the reactivity is a key to understanding of biological systems, where many processes in living cells occur in a confined space [9], as well as chemical systems, where various novel materials are created under spaciously confined conditions that give rise to unusual properties and chemical mechanisms [10].

Confined polymerization attracts research attention as a means of controlling the properties of polymers, such as molecular weight distribution [11,12,13], crystallinity [14,15,16], tacticity [13,17,18], and glass transition temperature [19,20,21]. Currently, the most studied type of confined polymerization is radical polymerization. For this type of polymerization the changes in the reactivity of monomers have been explained as the effects of confinement on the rates of the initiation [11] and termination [17,22]. Polycyclotrimerization of cyanate esters is another type of reaction for which the effect of confinement on both reactivity of the reactant monomer and product polymer has been well documented [23,24,25,26,27,28]. For these reactions, there have been no specific studies of the significant acceleration in terms of the reaction mechanisms. The increased reactivity of monomers has been attributed mostly to increased collision efficiency induced by the nanopore surface in the layer of the adsorbed monomer [25,26,28]. Although the effect of the surface silanols groups on the polymerization of cyanate esters has been invoked, it has never been studied in detail [29,30].

This current study focuses on the thermal polymerization of bisphenol E cyanate ester under both bulk and confined conditions. Silica colloidal crystals (SCCs) are used as a confining medium. To the best of our knowledge, this is the first study that makes use of SCC to explore confined chemical reactions. Chemical modification of SCC is used to reveal the influence of the nature of surface-grafted proton-donor groups on the reactivity of the confined monomer. The resulting reactions have been studied by differential scanning calorimetry (DSC), and their kinetics has been analyzed by means of the isoconversional methodology [31]. The analysis reveals that confinement causes a change of the reaction kinetics and mechanisms of polymerization that has been linked to the surface silanol groups participating in the polymerization process.

## 2. Materials and Methods

### 2.1. Materials

**Figure 1:**
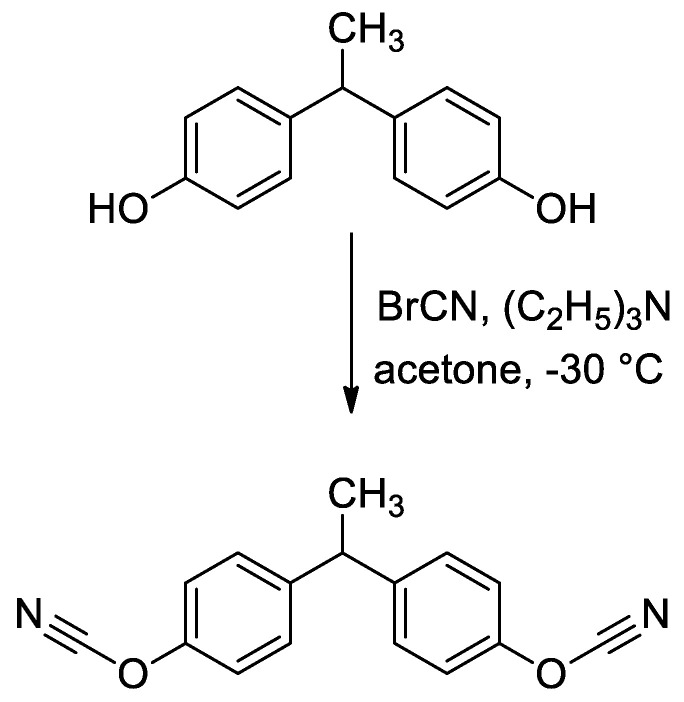
Scheme of synthesis of target cyanate ester.

Ammonium hydroxide solution (25% of NH_3_, TatChemProduct, Kazan, Russia), tetraethylorthosilicate (TEOS, >99.9%, ALDRICH Chemistry, Saint Louis, MI, USA), tetrabutylammonium hydroxide solution (40% in water, Fluka Analytical, Saint Louis, MI, USA), 1,3-propanesultone (98%, ALDRICH Chemistry, Saint Louis, MI, USA), bisphenol E (>98%, TCI, Zwijndrecht, Belgium), phosphorus pentoxide (99%, Vecton, Saint Petersburg, Russia), *n*-hexane (>98%, EKOS-1, Moscow, Russia), toluene (99.5%, EKOS-1, Moscow, Russia), and tetrahydrofuran (THF, >98%, ChemMed, Kazan, Russia) were purchased and used without additional purification. Bisphenol E cyanate ester of purity 99.5% was synthesized according to the previously described synthetic procedure (Figure 1) [32,33]. The purity of synthesized monomer was no less than 99.5% according to high-performance liquid chromatography (HPLC). Absolute ethanol was obtained by consecutive distillations of 96% ethanol over CaO and CaH_2_. Deionized water (18.2 MΩ) was obtained by Arium mini instrument (Sartorius, Goettingen, Germany).

### 2.2. Methods

Scanning electron microscopy (SEM) measurements were carried out using a Merlin field-emission high-resolution scanning electron microscope (Carl Zeiss, Oberkochen, Germany). An UP200Ht ultrasonic homogenizer (Hielscher Ultrasonics, Teltow, Germany) was used for all sonications. A MF48 centrifuge (AWEL, Blain, France) was used for all centrifugations. A LF-7/11-G1 furnace (LOIP, Saint Petersburg, Russia) was used for calcination and sintering. Contact angles were measured using a drop shape analyzer DSA100 (KRUSS GmbH, Hamburg, Germany); the water drop volume was 2 µL. High-performance liquid chromatography (HPLC) analyses were carried out with a Dionex Ultimate 3000 chromatograph (Thermo Fisher Scientific, Waltham, MA, USA) equipped with an UV detector and a Dionex Acclaim 120 chromatographic column (C18-bonded silica, 5 µm, 120 Å, 4.6 × 250 mm^2^). Nitrogen adsorption and desorption measurements were carried out at 77 K with an ASAP 2020 MP instrument (Micromeritics, Norcross, GA, USA). The specific surface areas of the SCCs were determined by applying the Brunauer–Emmett–Teller (BET) equation to adsorption data in a range of nitrogen relative pressure (P/P_0_) of 0.05–0.30, as recommended by IUPAC [34]. The total pore volume of the SCCs samples was measured at P/P_0_ = 0.999.

All calorimetric measurements were carried out using a heat flux DSC 3+ (Mettler-Toledo, Greifensee, Switzerland). Indium and zinc standards were used to perform temperature, heat flow, and tau-lag calibrations. The experiments were performed under an argon flow (80 mL min^−1^) at the heating rates of 2.5, 5.0, 7.5, 10.0, and 12.5 °C min^−1^ in 40 µL sealed aluminum pans. Because moisture could potentially affect polymerization, before sealing the samples were placed in 40 µL aluminum pans and kept in a vacuum desiccator containing P_2_O_5_ for 1 day to remove traces of water. The mass of the bulk cyanate ester samples for each run was ~1 mg. The mass of the SCCs samples loaded with the cyanate ester was taken to be ~5 mg that was the amount necessary to introduce ~1 mg of the cyanate ester into the available pores.

Preparation of silica spheres. Silica spheres with radii of 110 ± 4 nm were prepared by two-step controllable growth technique based on regrowth of silica seeds [35]. A detailed description of the procedure has been previously reported [36].

Preparation of sintered SCC (Sample A). SCCs were prepared by modified vertical deposition method based on isothermal heating evaporation-induced self-assembly (IHEISA) method [37]. Silica particles were dispersed in ethanol by sonication, and then a glass beaker containing the dispersion was placed in a homemade set-up at 79.8 °C. Obtained pieces of SCCs were carefully removed from the beaker’s walls and sintered at 850 °C for 12 h, and the desired temperature was achieved at a heating rate of 300 °C h^−1^. The particle size in SCCs was estimated by measuring 100 individual particles using SEM.

Surface modification of sintered SCCs. Rehydroxylation (Sample B): SCC synthesized with a mass of ~8 g was placed in a tetrabutylammonium hydroxide water solution with pH = 9.5 and T = 60 °C for 24 h [38]. Then, the sample was consistently washed by 1 M HNO_3_, methanol, deionized water, and acetonitrile and dried in vacuum at 200 °C for 2 h.

Introduction of 3-propylsulfonic acid groups (Sample C): ~1.5 g of rehydroxylated SCC was placed in 50 mL of toluene solution containing 3 mL of 1,3-propansultone and was refluxed for 24 h. Then, the samples were sequentially washed with toluene, THF, methanol, and hexane and dried in a furnace at 150 °C for 2 h.

Pore filling of SCCs. Loading of cyanate ester into colloidal crystals was carried as follows. A ~100 mg piece of a previously degassed colloidal crystal and a tenfold excess of the cyanate ester with respect to the pore volume of the SCC sample were placed into a vial and heated to 60 °C. Filling the pores of SCCs by the cyanate ester is driven by capillary forces and is readily monitored by the naked eye. An excess of the monomer on the piece of SCCs was carefully removed by fiber-free wipers. The extent of pore filling was estimated by as the difference in the mass of SCCs before and after impregnation and turned out to be close to 100% of pore volume for all samples.

## 3. Computations

The recommendations of the ICTAC Kinetic Committee were followed to evaluate the activation energy, preexponential factor, and the reaction model [39]. The extent of conversion, *α*, was determined as the partial area of the DSC peaks measured for polymerization of the cyanate ester. The effective activation energy, *E_α_*, was evaluated as a function of conversion with the aid of the flexible integral isoconversional method of Vyazovkin. The method affords eliminating a systematic error in *E_α_* when it varies with *α* [40]. This is accomplished by employing the so-called flexible integration that assumes the constancy of *E_α_* within a very narrow integration range, Δ*α*. The value of Δ*α* was taken as 0.01. Within each Δ*α*, *E_α_* is determined by finding a minimum of the function:

(1) $$\Psi \left(E_{\alpha }\right)=\overset{n}{\underset{i=1}{\sum }}\overset{n}{\underset{j\neq i}{\sum }}\frac{J\left[E_{\alpha },T_{i}\left(t_{\alpha }\right)\right]}{J\left[E_{\alpha },T_{j}\left(t_{\alpha }\right)\right]}$$

where

(2) $$J\left[E_{\alpha },T_{i}\left(t_{\alpha }\right)\right]\equiv \int_{t_{\alpha -\Delta \alpha }}^{t_{\alpha }}\exp\left[\frac{-E_{\alpha }}{RT_{i}\left(t\right)}\right]dt$$

and *n* is the number of the temperature programs. The integral was calculated by the trapezoid rule. The COBYLA non-gradient method from the NLopt library was utilized to determine a minimum of Equation (1). The uncertainties in the *E_α_* values were evaluated as described earlier [41].

The pre-exponential factor values were estimated by substituting the values of *E_α_* into the equation of the compensation effect.

(3) $$\ln A_{\alpha }=a+bE_{\alpha }$$

The parameters *a* and *b* were determined by fitting the pairs of ln*A_i_* and *E_i_* into Equation (4). The respective pairs were found by substituting the reaction models *f_i_*(*α*) into the linear form of the basic rate equation:

(4) $$\ln\left(\frac{d\alpha }{dt}\right)-\ln\left[f_{i}\left(\alpha \right)\right]=\ln A_{i}-\frac{E_{i}}{RT}$$

For each reaction model, the values of ln*A_i_* and *E_i_* were evaluated, respectively, from the slope and intercept of the linear plot of left-hand side of Equation (4) vs. the reciprocal temperature. In addition, the model $f\left(\alpha \right)=\alpha^{m}{\left(1-\alpha \right)}^{n}$ with five different pairs of *m* and *n* (*m* = 1, *n* = 1; *m* = 0.5, *n* = 1; *m* = 1, *n* = 0.5; *m* = 2, *n* = 1, *m* = 1, *n* = 2) was used for calculations of the pre-exponential factor values. This model was used because of its ability to imitate the autocatalytic kinetics which appears to be characteristic of the cyanate esters polymerization [42]. Furthermore, this model is similar to the Kamal reaction model [43], which has been used successfully to treat the kinetics of cyanate ester polymerization [32,44].

The isoconversional values of *E_α_* and ln*A_α_* were used to determine the rate constant. The rate constant was determined in the form of the Arrhenius plot (Equation (5)) [45].

(5) $$\ln k\left(T_{\alpha }\right)=\ln A_{\alpha }-\frac{E_{\alpha }}{RT}$$

Independently, the reaction model for the confined polymerization of bisphenol *E* cyanate ester was determined in the numerical form of Equation (6).

(6) $$g\left(\alpha \right)=\underset{\alpha }{\sum }A_{\alpha }\;J\left[E_{\alpha },T_{i}\left(t_{\alpha }\right)\right]$$

## 4. Results and Discussion

### 4.1. Synthesis and Characterization of SCCs Having Different Surface Chemistry

**Figure 2:**
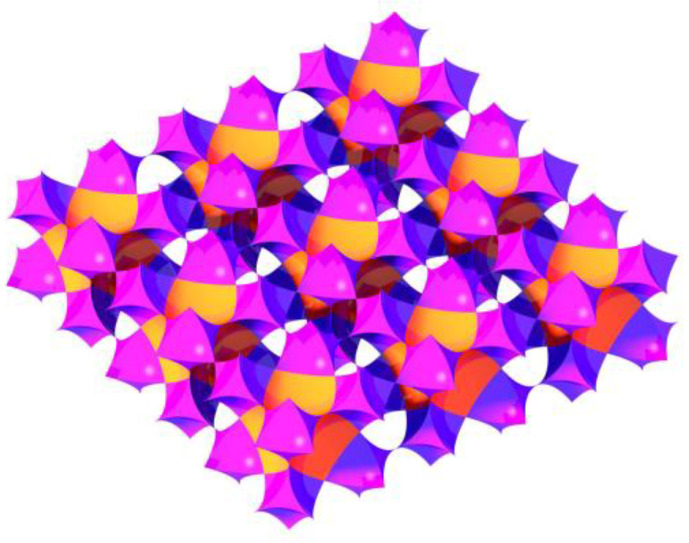
Interconnected octahedral (orange) and tetrahedral (purple) pores of silica colloidal crystal (SCC).

Studying the influence of confinement on chemical reactions usually makes use of porous media whose pores play the role of tiny reactors. The present study appears to be the first time when SCCs are used as a model porous medium. SCCs consist of silica spheres close-packed in a face-centered cubic arrangement, which implies existence of octahedral and tetrahedral pores. These two types of pores form an interconnected three-dimensional porous system (Figure 2).

Total porosity of an ideal close-packed face-centered cubic lattice is independent of the sphere size and equals to 0.2596, where share of octahedral pores is ~72% [36]. The porosity of real SCC may deviate to higher values because of the unavoidable polydispersity of the particles forming the crystal, which leads to different kinds of packing defects and results in reduced effectiveness of packing. Recently, it has been shown that despite the complex shape of the pores in SCCs they can be satisfactorily treated in terms of a spherical pore model [36]. It has been demonstrated that the effective diameters of the octahedral and tetrahedral pores equal, respectively, to 0.631 *× d* and 0.368 *× d*, where *d* is the diameter of silica spheres forming SCCs [36]. These estimates derive from the geometry of a face-centered cubic lattice and match closely the main modes of bimodal pore size distribution obtained from nitrogen adsorption data for mesoporous SCCs [46].

**Figure 3:**
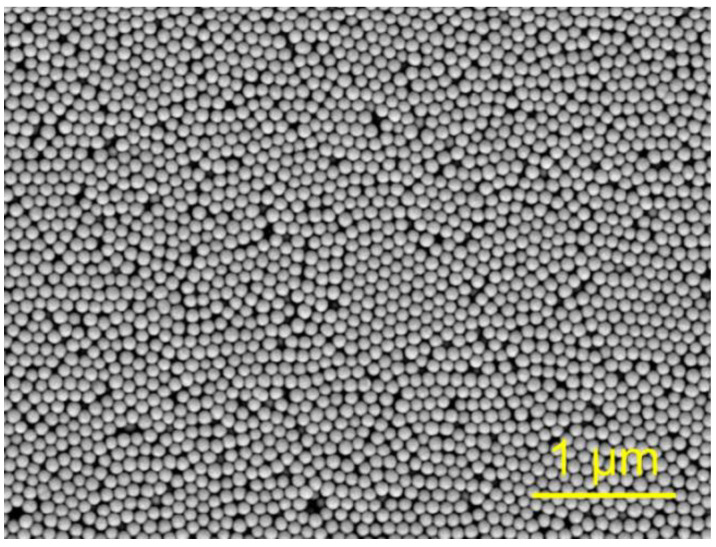
SEM image of (111) plane of the synthesized SCC made of 110 ± 4 nm silica spheres. Scale bar is 1 µm.

In the present study we have synthesized SCC consisting of 110 nm silica spheres. Scanning electron microscopy revealed the absence of macroscopic defects, e.g., cracks (Figure 3). On the other hand, some microscopic defects can be detected on (111) plane of SCC. Based on the aforementioned estimates, one can calculate the sizes of octahedral and tetrahedral pores of the synthesized SCC as ~70 and 40 nm, respectively.

**Figure 4:**
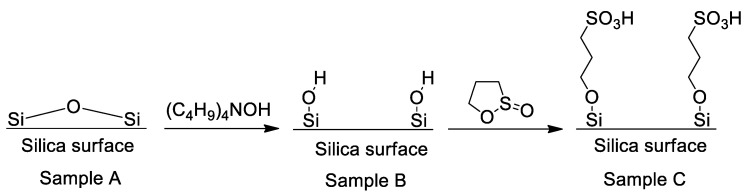
Surface modification of SCC.

Chemical modification of the synthesized SCC has been employed to alter the nature of the surface-grafted groups in order to assess their influence on the reactivity of the confined cyanate ester monomer. We have prepared three kinds of SCCs (A, B, and C) that have different surface chemistry. Namely, calcined crystals containing primarily siloxane surface fragments and surface silanols with the density ~0.6 silanols per nm^2^ (A), rehydroxylated crystals containing ~5 silanols per nm^2^ (B) [47], and the crystals, in which the silanols have been replaced by strong acid 3-propylsulfonic groups (C). According to the literature, sulfonation of rehydroxylated SCC by 1,3-propanesultone results in approximately half of the silanols to be replaced by the sulfonic groups [48]. Scheme of the modification is presented in Figure 4.

**Figure 5:**
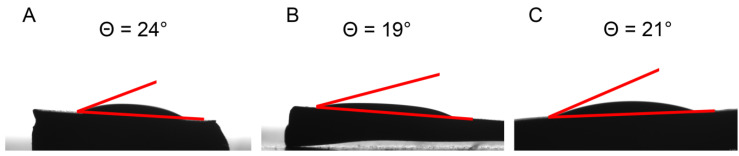
Water drop contact angle measurements of SCCs with different surface modifications (**A–C**).

Water drop contact angle measurements show how the modification of the SCCs affects the properties of the surface (Figure 5). It is seen that the SCCs with siloxane, silanols, and 3-propylsulfonic acid groups possess hydrophilic properties (Figure 5A–C).

Nitrogen adsorption measurements have provided textural parameters of synthesized porous materials (Table 1). It should be noted that the surface modification does not affect the specific surface area (S_BET_) and pore volume of the synthesized SCCs.

However, it is instructive to analyze the changes in the *C_BET_* parameter of the BET equation (Table 1), which characterizes the strength of adsorbate–adsorbent interaction (Equation (7)) [49]:

(7) $$C_{BET}=exp\left[\left(\Delta H_{des}-\Delta H_{vap}\right)/RT\right]$$

According to Equation (7), the value of the *C_BET_* parameter increases with increasing the difference between the enthalpy of adsorbate desorption from a monolayer (Δ*H_des_*) and the enthalpy of vaporization of the liquid adsorbate (Δ*H_vap_*). Thus, for a series of adsorbents with different surfaces larger values of *C_BET_* parameter corresponds to materials with higher polarity. The observed order of the surface polarity from the nitrogen adsorption measurements is consistent with the one from the water drop contact angle measurements (Figure 5).

### 4.2. Kinetics of Polymerization Studied by DSC

**Figure 6:**
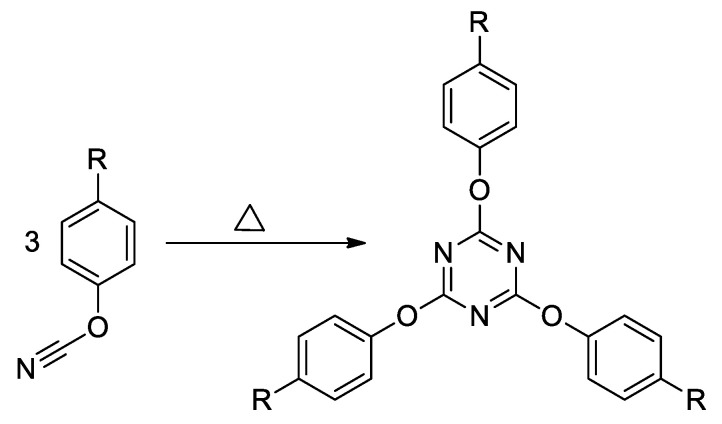
Scheme of cyclotrimerization of cyanate ester.

As the polymerization of cyanate ester is highly exothermic its progress is conveniently followed by DSC. The polymerization of cyanate esters occurs via formation of 1,3,5-triazine fragments from three cyanate groups (Figure 6).

**Figure 7:**
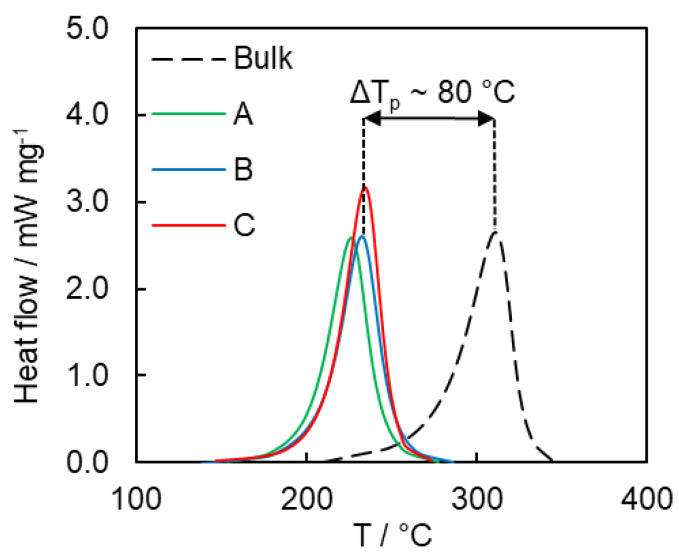
Heat flow curves for bisphenol E cyanate ester polymerization in bulk and nanoconfined states at 7.5 °C min^−1^. ΔT_p_ shows the magnitude of the difference in the peak temperatures of the respective DSC curves.

In the present study non-isothermal DSC measurements were used instead of isothermal ones to avoid possible incompleteness of the polymerization due to potential vitrification. The heat flow curves for polymerization of bulk and confined cyanate ester at a heating rate of 7.5 °C min^−1^ are presented in Figure 7. Clearly, confining the cyanate ester in the pores of any of the three types of SCCs results in significant acceleration of the polymerization rate as manifested by a significant shift of the reaction peak temperature to lower value (Figure 7).

**Figure 8:**
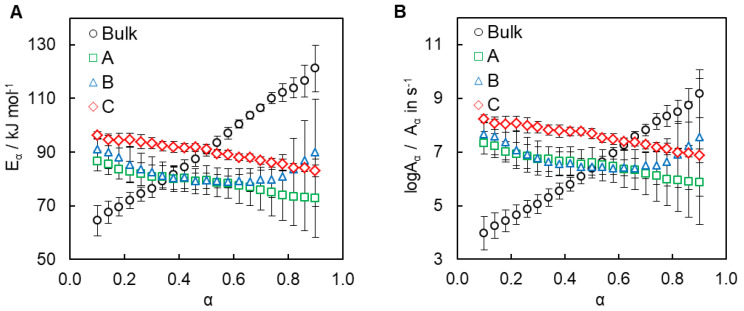
Dependencies of activation energy (**A**) and pre-exponential factor (**B**) as a function of conversion for polymerization of bulk and confined cyanate ester.

Average heats of the cyanate ester polymerization in the nanopores of SCCs are as follows; 657 ± 32 J g^−1^ (A), 714 ± 28 J g^−1^ (B), and 746 ± 28 J g^−1^ (C). These values are slightly lower than that of bulk polymerization (805 ± 42 J g^−1^). Further insights into the reactivity of the cyanate ester confined inside SCCs are obtained by isoconversional kinetic analysis. The isoconversional values of the activation energy *E_α_* and preexponential factor *A_α_* for bulk and confined monomer are displayed in Figure 8.

**Figure 9:**
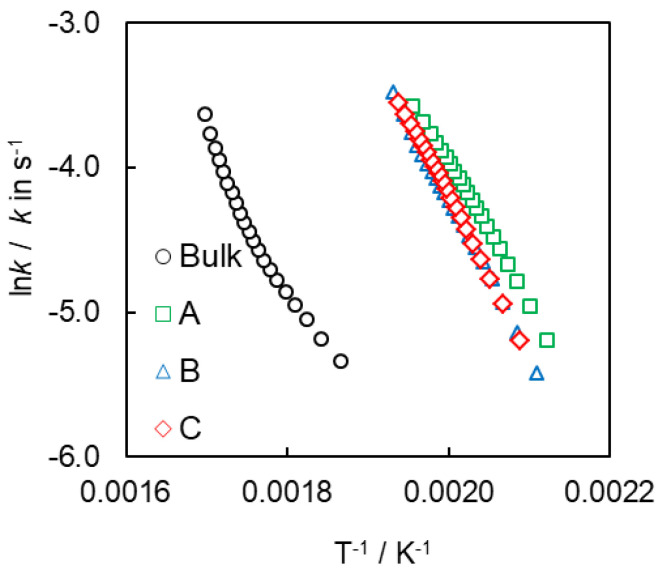
Arrhenius plots for polymerization of bulk and confined cyanate ester.

Comparing the isoconversional values of the effective activation energy (Figure 8A) of the confined processes one can see that the reactions in SCCs of types A and B are characterized by almost identical values of *E_α_*, whereas the reaction inside SCC of type B proceeds with larger activation energy that suggests that this process should be slower (Figure 8A). On the other hand, this reaction has larger values of the pre-exponential factor *A_α_* (Figure 8B), so it should be faster than two others. Combined effect of both Arrhenius parameters is reflected in the corresponding Arrhenius plots (Figure 9) that show that all confined systems have practically the same reactivity, just as observed from heat flow curves (Figure 7). Moreover, one can see a nonlinear dependence of ln*k* on T^−1^ for polymerization of cyanate ester in the bulk and an almost linear dependence of ln*k* on T^−1^ for polymerization in the hydrophilic pores of SCCs A–C.

Figure 8A shows that polymerization of bulk cyanate ester demonstrates a significant variation of the activation energy with conversion (variation of *E_α_* exceeds 60% of average *E_α_*), which is a sign of complex (i.e., multistep) nature of the polymerization process [50]. The pre-exponential factor values demonstrate a similar variation (Figure 8B).

A variation of the effective activation energy for bulk cyanate ester polymerization is expected, considering its complex mechanism that involves the formation of several intermediates [51]. Using different assumptions, several researchers have arrived at similar polymerization rate equations [44,52,53] that boil down to a model of two parallel reactions, one of which is autocatalytic (Equation (8)).

(8) $$\frac{d\alpha }{dt}=k_{1}{\left(1-\alpha \right)}^{n}+k_{2}\alpha^{m}{\left(1-\alpha \right)}^{n}$$

These two reactions are usually interpreted as a residual phenol-catalyzed process (*n*^th^-order reaction *k*_1_) and autocatalytic process catalyzed by the formed 1,3,5-triazine fragments [52,53]. It should be mentioned that the same equation, known as the Kamal model, is used for description of the kinetics of polymerization of epoxy-amine systems [43].

In the case of a process comprising two parallel reactions the effective activation energy should monotonically vary between the activation energies of the individual reactions according to Equation (9) [54]. This is exactly the trend observed for bulk polymerization.

(9) $$E_{\alpha }=-R{\left[\frac{\partial \ln\left(\frac{d\alpha }{dt}\right)}{\partial T^{-1}}\right]}_{\alpha }=\frac{A_{1}\exp(-E_{1}/RT)E_{1}+\alpha^{m}A_{2}\exp(-E_{2}/RT)E_{2}}{A_{1}\exp(-E_{1}/RT)+\alpha^{m}A_{2}\exp(-E_{2}/RT)}$$

To determine the kinetic parameters of the individual steps for bulk polymerization we have fitted Equation (10) to the dependence of the effective activation energy on temperature and conversion.

(10) $$E_{\alpha }=\frac{(A_{1}/A_{2})\exp(-E_{1}/RT)E_{1}+\alpha^{m}\exp(-E_{2}/RT)E_{2}}{\left(A_{1}/A_{2})\exp(-E_{1}/RT)+\alpha^{m}\exp(-E_{2}/RT\right)}$$

**Figure 10:**
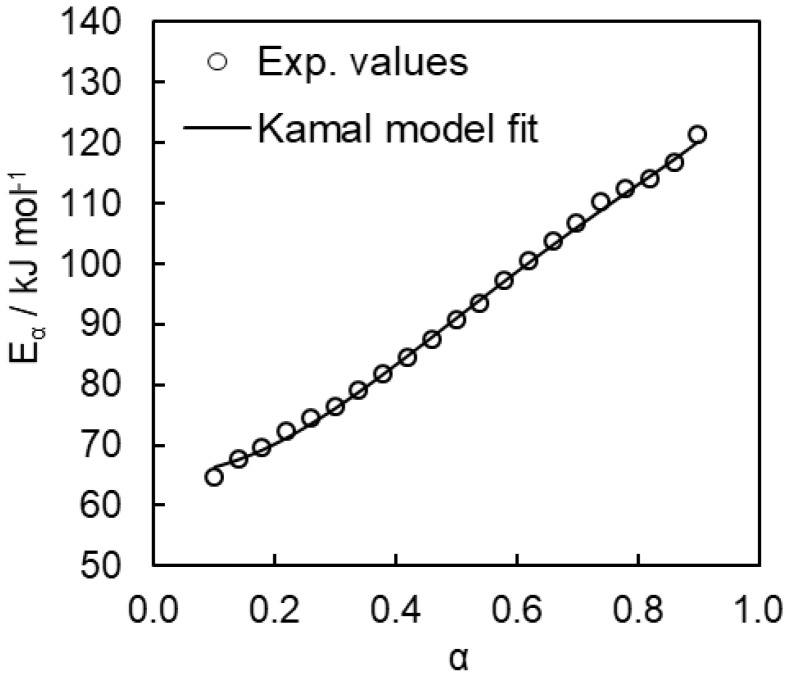
Fitting of the Kamal model to the experimental *E_α_* dependence for bulk polymerization.

This equation is a modified form of Equation (9) that has to be used instead of Equation (9) because the Equation (9) contains *A*_1_ and *A*_2_ as linear multipliers, which hampers their reliable evaluation [55]. Fitting Equation (10) to the experimentally determined dependence of the effective activation energy on temperature and conversion yields the *A*_1_/*A*_2_, *E*_1_, *E*_2_, and *m* parameters. An example of the fit is presented in Figure 10.

**Figure 11:**
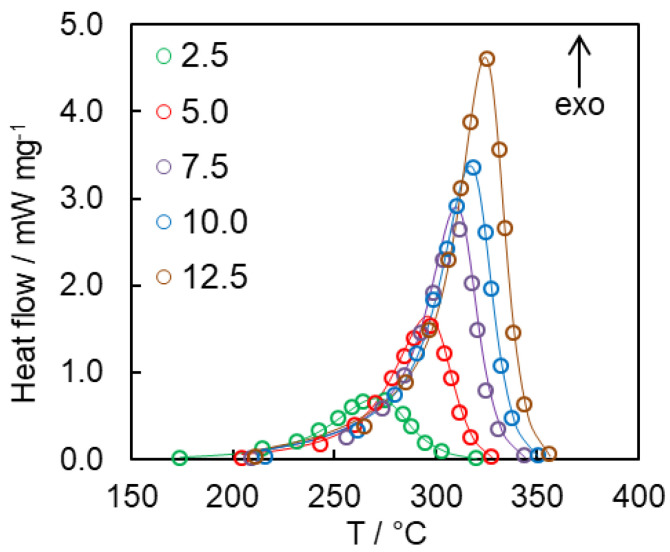
Comparison of the experimental heat flow curves (circles) with the calculated ones (lines) for bulk cyanate ester polymerization at different heating rates.

Evaluation of *A*_1_, *A*_2_, *n*, as well as refining the value of the *m* parameter has been the next step in our kinetic analysis. To do that, the *A*_1_, *A*_2_, *n*, and *m* parameters of Equation (8) have been evaluated by fitting this equation to the rate data for all heating rates. The *E*_1_ and *E*_2_ values have not been optimized in this fit. Their values have been kept equal to those found from fitting Equation (10). The results of these fittings recalculated for heat flow data are presented in Figure 11.

The resulting kinetics parameters for bulk polymerization of the cyanate ester are presented in Table 2. The parameters for bulk polymerization of the cyanate ester are similar to those reported for dicyanate esters of similar structure [32,44,53].

Analysis of the obtained *E_α_* and log*A_α_* dependencies for polymerization in the pores of SCCs (Figure 8) shows that acceleration of the polymerization at *α* < 0.5 is due to the fact that the pre-exponential factor values increase relative to bulk polymerization values. This agrees well with the literature data on the reaction of cyanate esters in silica nanopores, where acceleration of the reaction has been attributed to increased collision efficiency (higher pre-exponential factor values) in the monomer layer near the silica surface [25,26,28].

As seen in Figure 8A, polymerizations of the confined cyanate ester are characterized by significantly smaller variation of activation energy that does not exceed 20% of the respective average *E_α_*. Such variation can be considered insignificant so that the processes can be treated kinetically as a single step reaction [50]. We propose that one of the parallel reactions (i.e., *n*^th^-order or autocatalytic), included in the rate Equation (8), might be depressed in confinement conditions, so the polymerization process possesses single rate-limiting step.

**Figure 12:**
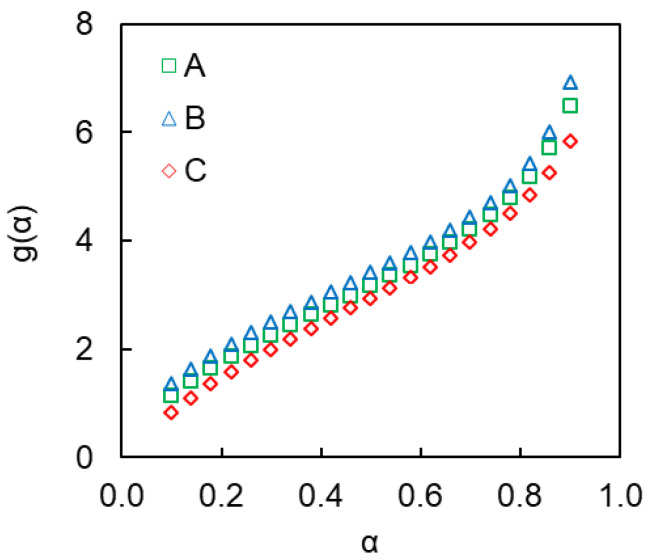
Experimental *g*(*α*) data for polymerization of cyanate ester confined to SCCs (A–C).

To figure out which reaction is depressed we have calculated the *g*(*α*) values according to Equation (6) for polymerization of the cyanate ester in the pores of the SCCs A–C (Figure 12). As one can see the *g*(*α*) plots for all three cases are nearly identical, so we can conclude that confined reactions proceed according similar reaction models.

Then we have fitted the expressions of *g*(*α*) for both aforementioned models (Equations (11) and (12)) to experimental data. It should be noted that unlike the *n*^th^-order reaction model

(11) $$g\left(\alpha \right)=\overset{\alpha }{\underset{0}{\int }}\frac{d\alpha }{{\left(1-\alpha \right)}^{n}}=\frac{1-{\left(1-\alpha \right)}^{1-n}}{1-n}$$

the autocatalytic model does not have an analytical form for *g*(*α*). Thus, the respective integral model has been evaluated numerically.

(12) $$g\left(\alpha \right)=\overset{\alpha }{\underset{0}{\int }}\frac{d\alpha }{\alpha^{m}{\left(1-\alpha \right)}^{n}}$$

**Figure 13:**
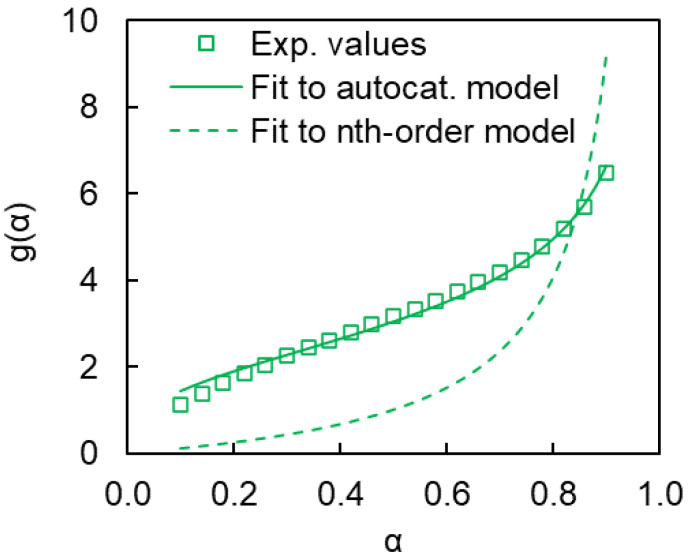
Fitting of the *n*^th^-order and autocatalytic models to the experimental *g*(*α*) dependence for polymerization of confined cyanate ester (SCC of type A).

It turns out that *n*^th^-order reaction model has produced fits of poor quality, whereas autocatalytic model has provided good results (Figure 13). Thus, one can conclude that under confinement the *n*^th^-order reaction is likely suppressed so that the overall reaction kinetics is controlled by an autocatalytic process (Equation (13)).

(13) $$\frac{d\alpha }{dt}=k_{2}\alpha^{m}\;{\left(1-\alpha \right)}^{n}$$

The calculated *m* and *n* parameters for all three studied reactions are very close to each other, and the average values of *m* and *n* are 0.67 ± 0.02 and 1.39 ± 0.02, respectively. The suppression of *n*^th^-order reaction, which corresponds to the residual phenol-catalyzed polymerization, might be explained by the preferential adsorption of these species onto the polar silica surface that diminishes their catalytic activity.

The obtained *m* and *n* values have been further refined by fitting the rate data to Equation (13) for all heating rates with the fixed activation energy value while varying the *A*, *m*, and *n* parameters. Previously determined averaged values of *A*, *m*, and *n* were used as initial values for optimization. The resulting values of the obtained kinetic parameters are presented in Table 2.

Table 2 shows that the reactions of the monomer in the pores of SCCs of the all three types are characterized by values of parameters of kinetic triplets, which are very close to each other and similar to those reported for catalytic polymerization of the same monomer [56].

It is instructional to compare the influence of phenols and proton-donor groups grafted onto the surface of SCCs. First, it is well known that an increase of the concentration of phenols results in an increase of the reaction rate, which is proportional to the phenol concentration raised to the power of 0.5–1.2 [57,58,59]. In contrast to the phenols an increase of the surface concentration of silanols by an order of magnitude from ~0.6 (sample A) to ~5 (sample B) silanols per nm^2^ as well as grafting of sulfonic acid groups of high acidity (sample C) does not appear to cause any appreciable increase in the acceleration of the polymerization process.

**Figure 14:**
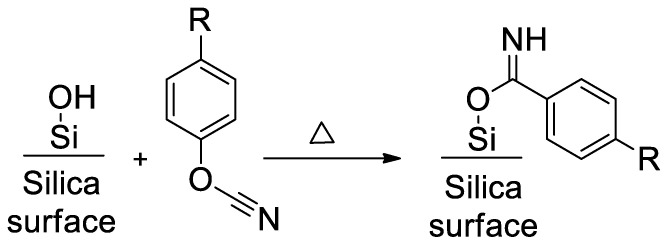
Scheme of formation of immobilized iminocarbonate.

Comparison of parameters of the autocatalytic reactions for the bulk and confined processes shows that the parameters of the reaction model (*m* and *n*) are the practically the same within the experimental error. Yet, the Arrhenius parameters (*E*_2_ and *A*_2_) differ significantly. The activation energy of the autocatalytic step in the bulk twice as large as the one for this step under confinement, but the pre-exponential factor value for the bulk step is larger by five orders of magnitude. The observed difference in the reactivity of the confined monomer can be explained by the different nature of the species promoting the autocatalytic process. Such species might be immobilized iminocarbonates formed by reaction of the cyanate ester monomer with silanol groups (Figure 14). Formation of such species was detected by FTIR during thermal polymerization of cyanate ester/silsesquioxane nanocomposites [29]. A characteristic absorption band at 1595 cm^−1^ has been assigned to hydrogen-bonded iminocarbonate groups [29]. Hydrogen-bonding is likely to increase the mobility of hydrogen linked to an iminocarbonate group, and thus to increase its catalytic activity in polymerization process [55]. As a result, the activation energy of the processes should be expected to decrease. 

**Figure 15:**
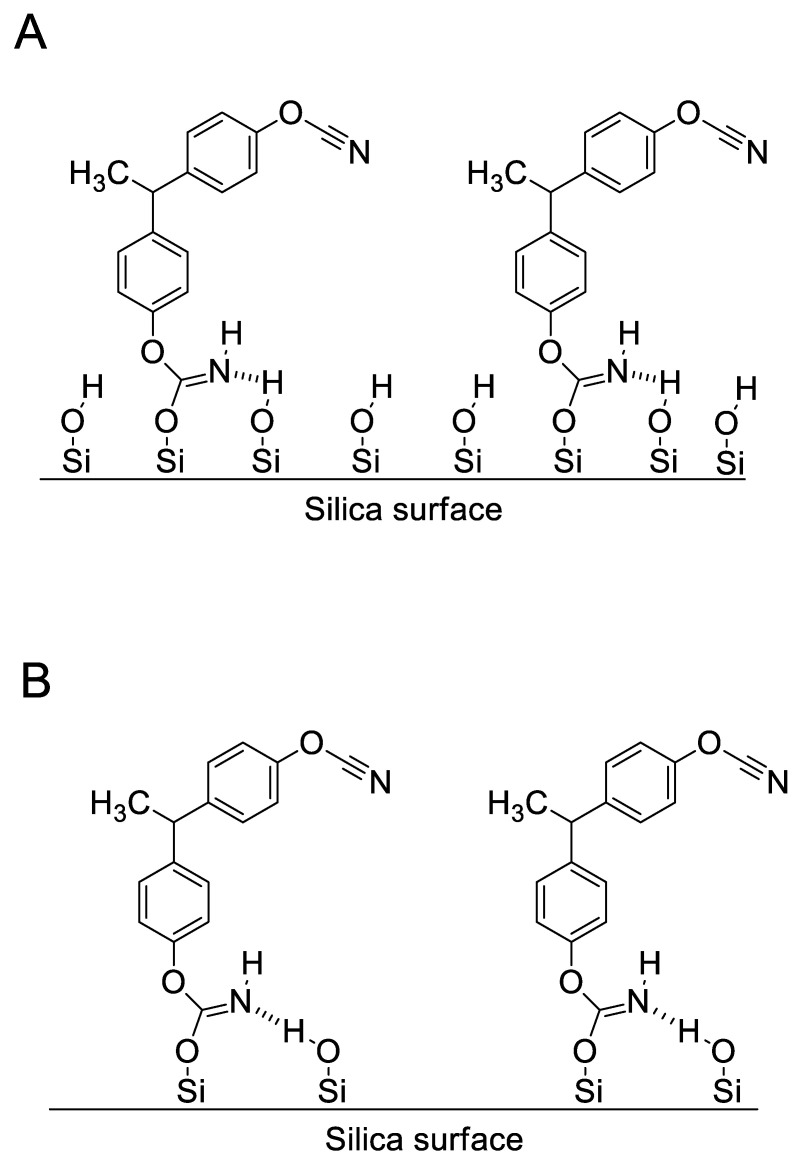
Illustration of immobilization of iminocarbonates on silica surfaces with high (**A**) and low (**B**) concentration of silanols.

On the other hand, the immobilization of iminocarbonate decreases its mobility, and thus should cause a decrease of the preexponential factor for the reaction it is involved in. Furthermore, because a fraction of the cyanate ester molecules is immobilized on the surface, they cannot participate in the cyclization process, which should result in a reduction in the reaction heats, as has been observed in the present study for the confined systems. Moreover, since the monomer molecules are quite bulky, the formation of iminocarbonate should hinder the reaction of neighboring silanols, which can be a reason for the absence of additional reaction acceleration inside the CSS having increased surface concentration of silanol groups (Figure 15).

In order to quantify the acceleration of confined reaction, we have introduced the isoconversional and isothermal acceleration factor *Z_α_*_,*T*_. This factor has a straightforward meaning of how many times the rate of the reaction under confinement is increased relative to that in the bulk at specific *T* and *α* (Equation (14)).

(14) $$Z_{\alpha ,\;T}=\;{\left(\frac{d\alpha }{dt}\right)}_{\alpha ,\;T}^{conf}/\;{\left(\frac{d\alpha }{dt}\right)}_{\alpha ,\;T}^{bulk}$$

**Figure 16:**
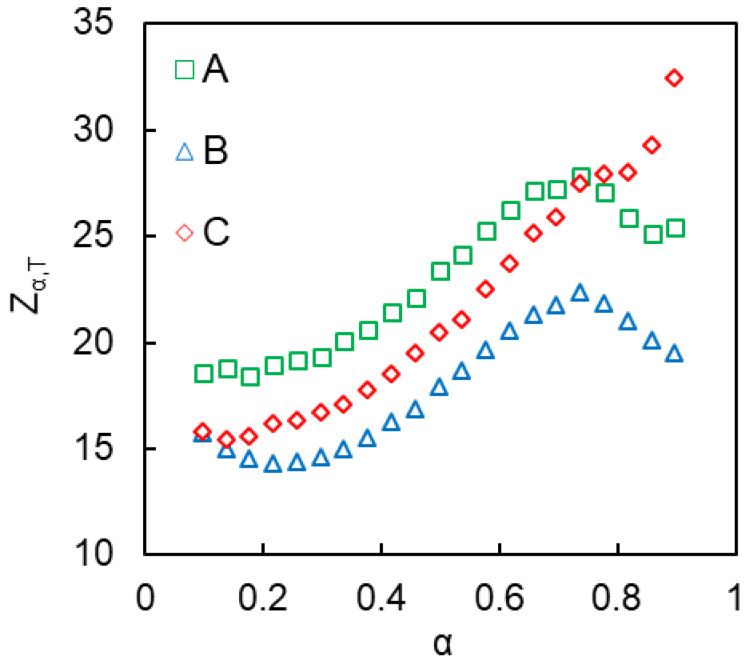
Variation of the isoconversional-isothermal acceleration factor *Z_α_*_,*T*_ with conversion at 250 °C.

Isoconversional values (*dα*/*dt*)*_α_* obtained from non-isothermal DSC runs cannot be directly used in Equation (14) since they correspond to different temperatures. Therefore, we recalculate the (*dα*/*dt*)*_α_* values from non-isothermal experiments to isothermal conditions. This is readily accomplished by using the technique of isoconversional predictions [31]. In the case when the isoconversional calculation is performed by integration over small segment of *α*, the respective equation takes the following form,

(15) $$t_{\alpha }=\underset{\alpha }{\sum }\frac{J\left[E_{\alpha },T_{i}\left(t_{\alpha }\right)\right]}{exp\left(-\frac{E_{\alpha }}{RT}\right)}$$

where *t_α_* is the time to reach the conversion *α* at isothermal temperature, *T*. The resulting set of the *α−t_α_* pairs is then differentiated numerically to determine (*dα*/*dt*)*_α_*_,*T*_ values. The latter are then substituted into Equation (14) to calculate the variation of *Z_α_*_,*T*_ factor with conversion. The resulting values of *Z_α_*_,*T*_ at *T* = 250 °C are shown in Figure 16. This temperature was chosen as a reference because all studied systems react with measurable rates at that temperature (see Figure 6). Based on the *Z_α_*_,*T*_ values we can conclude that the confined reactions proceed about 15–30 times faster than polymerization in the bulk.

## 5. Conclusions

We have studied the kinetics of polymerization of the cyanate ester monomer confined to the hydrophilic pores of SCCs. The crystal surface has been modified to probe the effect of the concentration of silanol groups and acidity of grafted 3-propylsulfonic groups. Significant acceleration of polymerization has been observed in all three types of SCCs. Kinetic analysis has revealed that the bulk polymerization follows the model of two parallel reactions, namely, *n*^th^-order and autocatalytic. Confining the monomer to the hydrophilic pores has simplified the polymerization kinetics making it essentially a single-step one. Kinetic analysis of the confined polymerization suggests that *n*^th^-order reaction, corresponding to phenol-catalyzed polymerization, is suppressed likely due to adsorption of residual phenols by the polar silica surface. Kinetic parameters of the confined autocatalytic polymerization have been similar for all three types of SCCs so that no significant effect of the concentration and proton acidity of the surface-grafted groups has been found. On the other hand, the activation energy of the confined autocatalytic polymerization has been determined to be about two times smaller than for the bulk process. Moreover, the confined process has demonstrated a decrease in the pre-exponential factor and the reaction heat. These effects have been explained by the formation of iminocarbonates that are immobilized on the silica surface but possess increased catalytic activity due to hydrogen-bonding with residual silanol groups. In addition, we have introduced the acceleration factor *Z_α_*_,*T*_ that permits to compare conveniently the reactivity of compounds reacting in different temperature ranges.

## Figures and Tables

**Table 1:** Textural parameters of synthesized SCCs A–C.

	S_BET_, m^2^ g^−1^	Pore Volume, cm^3^ g^−1^	C_BET_
A	34 ± 1	0.26 ± 0.01	103 ± 15
B	33 ± 1	0.26 ± 0.01	182 ± 53
C	34 ± 1	0.25 ± 0.01	135 ± 33

**Table 2:** Calculated kinetic parameters for bulk and confined polymerization of cyanate ester.

	*E*_1_/kJ mol^−1^	log*A*_1_/*A* in s^−1^	*E*_2_/kJ mol^−1^	log*A*_2_/*A* in s^−1^	m	n
Bulk	65 ± 1	3.1 ± 0.1	154 ± 3	12.0 ± 0.1	0.9 ± 0.3	1.2 ± 0.1
A	-	-	79 ± 6	6.6 ± 0.1	1.0 ± 0.1	1.4 ± 0.1
B	-	-	83 ± 6	6.9 ± 0.1	1.0 ± 0.1	1.4 ± 0.1
C	-	-	90 ± 4	7.5 ± 0.1	0.9 ± 0.1	1.1 ± 0.1
